# Supplementary material for: Operationalizing the 2018 World Cancer Research Fund/American Institute for Cancer Research (WCRF/AICR) Cancer Prevention Recommendations: A Standardized Scoring System
Source: Nutrients. 2019 Jul 12;11(7):1572. doi: 10.3390/nu11071572 (PMC6682977; doi:10.3390/nu11071572)
Supplement: Supplementary file 1 [file nutrients-11-01572-s001.pdf]

Food items	aUPFs	All UPFs
Sour cream (regular, low-fat, fat-free)	x	x
Cream cheese (regular, low-fat, fat-free)	x	x
Ice cream, regular	x	x
Frozen yogurt, ice milk	x	x
Beef burgers (lean, regular)		x
Hot dogs (regular, low-fat, turkey)		x
Fried chicken (light/dark meat, with/without skin)	x	x
Fried fish	x	x
Creamed soups	x	x
English muffins, bagels, white breads and rolls, crackers (high-fat, low-fat), tortillas	x	x
Cornbread/muffins, biscuits, quick breads, donuts, sweet rolls, pancakes, waffles, french toast	x	x
Ready-to-eat cereal (highly fortified, other)	x	x
Cookies, brownies, cakes (regular, low-fat)	x	x
Pies (fruit, cream/custard, pumpkin, sweet potato, other)	x	x
Chips (potato,corn,other, regular, low-fat), popcorn	x	x
Rice, grains (NFA)	x	x
Pasta (NFA), lasagna, ravioli, shells, etc.	x	x
Macaroni and cheese, pizza, fried potatoes	x	x
Gravy	x	x
Potato salads	x	x
Margarine (stick, tub, diet), margarine/butter blend, and butter- on bread, pancakes, waffles, potatoes, vegetables, and other uses	x	x
Salad dressing (regular, low-fat, nearly non-fat)	x	x
Mayonnaise (regular, diet, fat-free)- on sandwiches and salad	x	x
Vegetable shortening, lard, fatback bacon fat	x	x
Candy (chocolate and non-chocolate)	x	x
Presweetened tea (regular, decaf), soft drinks (regular, decaf), fruit drinks (regular)		x
Diet, presweetened tea and soft drinks (caffeinated, decaffeinated), diet fruit drinks	x	x
Saccharine, aspartame (in coffee or tea)	x	x

Figure S1. Ultra-processed foods (UPFs) included to create the adapted ultra-processed food variable (aUPF) in the NIH-AARP Diet and Health Study dataset

Table S1. Distribution of percent total energy from UPFs vs. aUPFs in the NIH-AARP Diet and Health Study (N=545,825)

Tertiles	N	Median *(%)*	Minimum *(%)*	Maximum *(%)*
Including all UPFs[1]				
T1	181,941	29.935	0	36.738
T2	181,942	42.342	36.739	48.003
T3	181,942	55.18	48.003	99.097
Including aUPFs only[2]				
T1	181,941	26.942	0	33.031
T2	181,942	37.958	33.032	42.930
T3	181,942	49.246	42.930	99.097

aUPFs, adapted ultra-processed foods; UPFs, ultra-processed foods from the NOVA classification system [28]

[1] Ultra-processed foods (UPFs) were defined with guidance from the NOVA classification system

[2] Sugar-sweetened drinks and red and processed meats were removed from the UPF variable to create the adapted UPF variable (aUPF)
